# Designing artificial circadian environments with multisensory cares for supporting preterm infants’ growth in NICUs

**DOI:** 10.3389/fnins.2023.1152959

**Published:** 2023-08-24

**Authors:** Takeshi Arimitsu, Rika Fukutomi, Mayuko Kumagai, Hayato Shibuma, Yoko Yamanishi, Kei-ichi Takahashi, Hirotaka Gima, Yoshitaka Seto, Hiroyuki Adachi, Hirokazu Arai, Masakatsu Higuchi, Shohei Ohgi, Hidenobu Ohta

**Affiliations:** ^1^Department of Pediatrics, Keio University School of Medicine, Tokyo, Japan; ^2^The Japan Developmental Care Study Group, School of Rehabilitation Sciences, Seirei Christopher University, Hamamatsu, Japan; ^3^Section of Pediatric Nursing, Graduate School of Nursing Science, St. Luke's International University, Tokyo, Japan; ^4^Department of Nursing, Akita University Graduate School of Medicine, Akita, Japan; ^5^Department of Rehabilitation, Yamagata Saisei Hospital, Yamagata, Japan; ^6^Department of Occupational Therapy, Faculty of Health Sciences, Tokyo Metropolitan University, Tokyo, Japan; ^7^Department of Occupational Therapy, Akita University Graduate School of Medicine, Akita, Japan; ^8^Department of Physical Therapy, Faculty of Health Sciences, Tokyo Metropolitan University, Tokyo, Japan; ^9^Maternity and Perinatal Care Center, Hokkaido University Hospital, Sapporo, Japan; ^10^Department of Pediatrics, Akita University Graduate School of Medicine, Akita, Japan; ^11^Department of Neonatology, Akita Red Cross Hospital, Akita, Japan; ^12^Department of Occupational Therapy, Faculty of Health and Medical Science, Teikyo Heisei University, Tokyo, Japan; ^13^Department of Physical Therapy, School of Rehabilitation Sciences, Seirei Christopher University, Hamamatsu, Japan; ^14^Department of Sleep-Wake Disorders, National Institute of Mental Health, National Center of Neurology and Psychiatry, Tokyo, Japan; ^15^Department of Psychiatry, Asai Hospital, Chiba, Japan

**Keywords:** designing artificial environments, NICUs, preterm infants, circadian rhythm, multisensory cares

## Abstract

Previous studies suggest the importance of stable circadian environments for fetuses to achieve sound physiology and intrauterine development. This idea is also supported by epidemiological and animal studies, in which pregnant females exposed to repeated shifting of light–dark cycles had increased rates of reproductive abnormalities and adverse pregnancy outcomes. In response to such findings, artificial circadian environments with light–dark (LD) cycles have been introduced to NICUs to promote better physical development of preterm infants. Such LD cycles, however, may not be fully effective for preterm infants who are less than 30 weeks gestational age (WGA) since they are too premature to be adequately responsive to light. Instead, circadian rhythmicity of incubated preterm infants less than 30 WGA may be able to be developed through stimulation of the non-visual senses such as touch and sound.

## Introduction

British author Aldous Huxley used his genius to predict future Neonatal Intensive Care Units (NICUs) in his 1932 fictional novel ‘Brave New World’ (Huxley, 1932). In the NICU he describes, human embryos are incubated in transparent bottles under a dim red light similar to the red colored sunshine we see when we close our eyelids outside on a summer afternoon. The NICU described in his novel also has an artificially-controlled environment high in temperature and humidity, like in some tropical areas.

Huxley’s description of temperature and humidity for the fetuses as being “tropical” matches well with the present settings for preterm infants in modern incubators. In Japan, preterm infants with a weight of <1,000 g are usually accommodated in incubators at a temperature of approximately 34–35°C and a humidity of 60–70% for their first week of life after birth. However, the lighting conditions he states – a constant dim red light – differs from the light–dark cycle which several circadian studies and the American Academy of Pediatrics (AAP) and American Colleges of Obstetrician and Gynecologists (ACOG) recommend for preterm infants in NICUs to encourage circadian entrainment (White, 2013; AAP Committee on Fetus and Newborn and ACOG Committee on Obstetric Practice, 2017). More recent studies have suggested that appropriate multisensory exposures to preterm infants in the NICU may also improve their postnatal development (Pineda et al., 2021; Schmidt Mellado et al., 2022). However, further research on achieving sound physiology and extrauterine development of preterm infants though NICU environment is still required. This review focuses on constructing possibly better NICU environments, particularly paying attention to circadian effects on the body growth of preterm infants.

## Does biological clock influence fetal growth?

Among past literature, only two types of studies seem to report the possibility that biological clocks affect fetal body growth. One is an animal study in which rat fetal growth was measured in pregnant mother rats exposed to different lighting conditions. According to the study, the fetal weights in pregnant mother rats exposed to constant light were decreased compared to the fetal weights in pregnant rats exposed to light–dark (LD) cycles (Mendez et al., 2012). In LD conditions, the mother rats had circadian peaks of melatonin at night while the mothers in constant light lost their melatonin circadian rhythms. Moreover, restoration of maternal melatonin rhythmicity via night-time melatonin injection in mother rats exposed to constant light rescued their fetal growth restriction. The authors suggest that maternal circadian rhythm of melatonin leads to fetal circadian rhythm of corticosterone in the adrenal gland and that fetal corticosterone rhythm then contributes to fetal growth. The physiological mechanism linking fetal corticosterone rhythm and body growth, however, remains unclear.

The other type of study is clinical studies in which weight gains of human preterm infants were found to be better in LD conditions than in constant light or dark conditions (Watanabe et al., 2013; Morag and Ohlsson, 2016). Preterm infants are born before term and raised in incubators with a 24-h monitoring system, and an artificial ventilator or an ECMO (extra-corporeal membrane oxygenator), depending on their medical situation. The 24-h monitoring systems tell us that no circadian rhythms exist in the heart rate, respiratory rate, oxygen saturation or EEG of preterm infants (Glotzbach et al., 1995) in non-LD conditions. This is in contrast to human fetuses, who have been reported to have circadian rhythms in heart rates and fetal movements at around 20–22 weeks gestational age (WGA) (de Vries et al., 1987; Lunshof et al., 1998). Previous studies suggest that the circadian systems of fetuses are still premature and dependent on maternal circadian signals such as hormonal or nutritional signals, or on signals through maternal physical activity (Seron-Ferre et al., 2007; Watanabe et al., 2013). The researchers suggest a possibility that LD conditions support the immature biological clocks of preterm infants and premature animals by directly affecting their suprachiasmatic nuclei (SCN) through their photoreceptors, the intrinsically-photosensetive RGCs (ipRGCs) and/or rods, in their eyes, leading to stable circadian rhythms within the SCN (Sekaran et al., 2005; Sernagor, 2005; Schmidt et al., 2008; Van Gelder, 2008; Watanabe et al., 2013; Hazelhoff et al., 2021; Caval-Holme et al., 2022; Polese et al., 2022). Mouse studies have also revealed that the circadian systems of the SCN are disrupted by constant light at the cellular level (Ohta et al., 2005, 2006). The physiological mechanism linking LD conditions and body growth in preterm infants, however, remains unclear and further research in this area is still required.

If these animal and clinical studies present correct results, at least the following three hypotheses can be derived. First, the clinical studies suggest that the circadian mechanism for the growth of preterm infants does not need to depend on maternal signals. Since human preterm infants in incubators, who are completely separated from their mother, are able to achieve appropriate growth in an LD cycle, it can be assumed that maternal signals are not absolutely necessary for the appropriate growth of preterm infants.

Second, clinical studies also suggest that melatonin does not directly contribute to the circadian mechanism for the growth of preterm infants. In humans, melatonin does not have circadian rhythms at birth - at around 40 WGA - and do not exist until approximately 3 months of age (Kennaway, 2000; Rivkees, 2003). Although nighttime increase in melatonin concentration of breast milk in a circadian manner has been reported to occur 9 days after delivery and may support development of the circadian rhythms of infants (Illnerová et al., 1993), their circadian rhythms in sleep–wake only start to be observed after approximately 2 months of age (Rivkees, 2003). Since preterm infants do not have circadian rhythms of melatonin, circadian signals from external or internal factors, such as lighting, nutrition or other hormones must affect the biological clocks of preterm infants. Detection of these, however, is difficult to achieve, as it would require blood samples to be taken from infants every three to 4 h for examination, which is ethically unacceptable (Dupont-Thibodeau and Janvier, 2016).

Third, the animal study with melatonin injections into pregnant rats (Mendez et al., 2012) suggests that the SCN or the organ biological clocks, which modulate circadian phases through melatonin receptors, contribute to the circadian mechanism for the body growth of preterm infants, although melatonin itself does not seem to directly affect the body growth of preterm infants (Gombert and Codoñer-Franch, 2021). To date, two membrane-bound melatonin receptors have been identified and characterized - MT1 and MT2. MT1 receptors are expressed in the brain, cardiovascular system (including peripheral blood vessels, aorta and heart), immune system, testes, ovary, skin, liver, kidney, adrenal cortex, placenta, breast, retina, pancreas and spleen (Dubocovich and Markowska, 2005; Slominski et al., 2005; Fischer et al., 2008; Pandi-Perumal et al., 2008; Slominski et al., 2008). In the brain, the MT1 receptor is predominantly found in the SCN, hypothalamus, cerebellum, hippocampus, substantia nigra and ventral tegmental area (Pandi-Perumal et al., 2008). MT2 has been found in the brain (SCN, hypothalamus and pituitary), retina, immune system, blood vessels, testes, kidney, gastrointestinal tract, mammary glands, adipose tissue, and the skin (Reppert et al., 1995; Roca et al., 1996; Dubocovich and Markowska, 2005; Slominski et al., 2005).

These three hypotheses lead us to a possible conclusion that the retina, the SCN, and the adrenal gland, each of which can be affected by both light and melatonin, may each possibly be part of the pathway for the circadian mechanism for the growth of preterm infants.

Regarding the retina and SCN, the retina has a wide range of efferent visual and non-visual pathways to the brain. The visual pathways, which contribute to both image formation and light–dark detection, are based on the nerve innervations from the rods and cones in the retina. The visual pathways have direct projections to the dorsal lateral geniculate nucleus (dLGN) and superior colliculus (SC). Non-visual pathways, which contribute to only light–dark detection, are composed of intrinsically-photosensetive RGCs (ipRGCs). The non-visual pathways have direct projections to the suprachiasmatic nucleus (SCN), supraoptic nucleus (SON), subparaventricular zone (SPZ), ventral lateral preoptic area (VLPO), lateral hypothalamic area (LH), anterior hypothalamus (AH), medial amygdaloid nucleus (MA), habenula (Hb), bed nucleus of the stria terminalis (BST), dorsal lateral geniculate nucleus (dLGN), ventral lateral geniculate nucleus (vLGN), intergeniculate leaflet (IGL), periaqueductal gray (PAG), olivary pretectal nucleus core (OPN), and superior colliculus (SC). Via the SCN, the non-visual pathways also have indirect projections to the paraventricular nucleus (PVN), dorsomedial hypothalamus (DMH), locus coeruleus (LC), ventral tegmental area (VTA), and septal area (Sept) (Dacey et al., 2005; Hattar et al., 2006; Brown et al., 2010; Noseda et al., 2010; Milosavljevic, 2019; Aranda and Schmidt, 2021).

No direct efferent pathway from the retina to the visual or frontal cortex has been anatomically detected (Gaillard et al., 2013). A human EEG study, however, using light stimulus on blind people, in whom no visual pathway but only the non-visual pathway is supposed to function, suggests that the non-visual pathway may have possible projections to the visual cortex (Vandewalle et al., 2018). A mouse study also indicates that the non-visual pathway can affect cortical synaptogenesis at early developmental stages via the SON and PVN by light-dependent oxytocin secretion (Hu et al., 2022). The ipRGCs-dependent non-visual pathway contributes to various physiological functions such as entrainment of circadian rhythms (Panda et al., 2002; Ruby et al., 2002), sleep–wake regulations (Lupi et al., 2008), pupil constriction (Hattar et al., 2003; Watanabe et al., 2013; Adhikari et al., 2015; Ikeda et al., 2015), alertness (Milosavljevic, 2019), mood (LeGates et al., 2012; Fernandez et al., 2018), learning (Fernandez et al., 2018), and visual perception (Aranda and Schmidt, 2021). In particular, in terms of circadian rhythms and sleep–wake regulation, the SCN directly controls circadian rhythms and the IGL also indirectly entrains circadian rhythms (Hatori and Panda, 2010; Aranda and Schmidt, 2021). In addition, the lateral and posterior hypothalamus, the VLPO, the Hb, and the SPZ are believed to mediate the effects of light on the hypothalamic regulation of sleep, behavior, and other physiological functions (Hatori and Panda, 2010; Aranda and Schmidt, 2021). These facts imply that the retina and/or SCN may contribute to the circadian mechanisms for the body growth of preterm infants.

The adrenal cortex is another candidate partial pathway since light information is conveyed from the SCN to the adrenal gland via the SCN-sympathetic nervous system (Ishida et al., 2005) and MT1 receptors are also detected in the adrenal cortex in adult animals (Torres-Farfan et al., 2003; Richter et al., 2008). Like the development of melatonin circadian rhythms, however, no clear cortisol circadian rhythms are detected in humans until approximately 1 month of age (Ivars et al., 2015, 2017), suggesting that cortisol may not contribute to the circadian mechanism for the body growth of preterm infants until that time.

Overall, the above suggests that we might be able to improve the body growth of preterm infants by providing the SCN and retina with artificial circadian signals, such as a light–dark (LD) cycle, or nighttime-injected melatonin.

## Discussion

### Is a light–dark cycle beneficial for the body growth of preterm infants? – from an implication of intrauterine circadian effects on fetal growth

The concept of a stable circadian uterine environment having positive effects on fetal development is also supported by clinical studies in which pregnant females exposed to repeated shifting of their LD cycle had increased rates of reproductive abnormalities and adverse pregnancy outcomes such as preterm delivery and particularly low birth weight of offspring. Several epidemiological studies on humans have identified associations between shift work or repeated travel across time zones and reduced fertility (Bisanti et al., 1996) as well as negative pregnancy outcomes, including increased incidence of low birth weight, preterm birth and miscarriage (Cone et al., 1998; Aspholm et al., 1999; Knutsson, 2003). However, whether these adverse outcomes are due to circadian dysregulation or some other lifestyle factor associated with shift work has not been clearly determined.

Also, the study mentioned earlier in which pregnant mother rats were exposed to different lighting conditions reported that exposure of pregnant rats to constant light, which disrupts the circadian environment for fetuses, induced intrauterine growth restriction and also lowered corticosterone production in fetal adrenal glands (Mendez et al., 2012). Other studies using rodent models indicated that repeated shifting of the light–dark cycle of pregnant rats and mice increased rates of miscarriage and induced hyperleptinemia and hyperinsulinemia in offspring lasting into adulthood (Varcoe et al., 2011; Summa et al., 2012). Shifts in rats’ LD cycle are known to transiently disrupt normal phase relationships between the SCN and peripheral biological clocks (Yamazaki et al., 2000), thus desynchronized circadian rhythms, either between central and peripheral maternal tissues or between maternal and fetal tissues (or both), may in part contribute to the adverse effects of chronic environmentally-mediated circadian disruption on pregnancy outcomes.

### When should a light–dark cycle be started for preterm infants?

In theory, an LD cycle might best be introduced to preterm infants at around 26–30 WGA, when the eyes of preterm infants can start to perceive light. Robinson and Fielder (1990) reported that preterm infants can respond to light from 30 weeks postmenstrual age (WPA), equivalent to 30 WGA, based on measurements they had made of the pupillary light reflex (PLR). Another study by Hao and Rivkees (1999) using a baboon model indicated that the SCN in the baboon fetus of an age corresponding to human 24 weeks conceptional age (WCA), approximately equivalent to 26 WGA in the human fetus, was already responsive to light stimuli. The data suggest that preterm infants might start to respond to light from around 26–30 WGA.

In practice, however, an LD cycle would best be introduced to preterm infants just after birth without considering when preterm infants start to perceive light. The reason for this is the large individual difference in when preterm infants start to respond to light. In a study of the development of PLR, Robinson and Fielder reported that the earliest that some preterm infants start to respond to light is at 30 WGA, while others start to respond to light as late as 34 WGA (Robinson and Fielder, 1990), indicating that the onset of the PLR could vary up to 5 weeks between individual preterm infants. It is quite time-consuming and stress inducing to examine preterm infants’ PLR every week to confirm whether the infants have started to respond to light. Instead, if the NICU is always set to an LD cycle, each preterm infant will automatically entrain their biological clock to the LD cycle as their biological clocks start to respond to light without any need for regular PLR checks.

Strict light control of LD cycles in NICUs, however, might not provide the best benefit for preterm infants of <30 WGA being intensively treated for serious medical conditions just after birth. Since they are in a critical physiological state and have minimal sensitivity to light because of the immaturity of their photoreceptors, a constantly lit environment would allow easier performance of intensive medical treatments and cares, operation of medical equipment and observation of the infants’ physiological condition and behavior without any major adverse effect on the rhythm of their biological clock.

### What type of light- dark cycle should be provided for preterm infants?

To design appropriate lighting conditions for preterm infants in NICUs, we have to take into account at least the following three factors: duration, light intensity, and light wavelengths of light/dark periods.

#### What durations of light/dark periods are appropriate for NICUs?

Previous studies suggest that a 12 L:12D LD cycle would be safe as a NICU lighting cycle. However, no clinical study in human neonatal physiology has investigated an optimum ratio of light/dark period duration for NICUs. Adult studies, however, have demonstrated that humans maintain stable circadian rhythms of behavior and hormonal secretions by adapting to different day lengths (photoperiods) between short day (8 h light period: 16 h dark period (8 L:16D)) and long day (14 L,10D). Compared to the long-day photoperiod, the short-day photoperiod provides us with longer sleep durations, longer nocturnal periods of low rectal temperature, longer nocturnal periods of active melatonin secretion, and higher levels of prolactin and cortisol secretion (Thomas et al., 2002).

Animal studies have also examined the effects of photoperiod during early infancy on development of circadian clocks and other physiological parameters. Ciarleglio et al. (2011) found that mice reared in long days (16 L:8D) exhibited a shorter free-running period of locomotor activity rhythms compared to mice reared in short days (8 L:16D). This is consistent with their results from Per1, a clock gene expression in the SCN, in which perinatal exposure to long days (16 L:8D) induced a narrower Per1 expression waveform and shorter rhythm period compared to short-day (8 L:16D) exposure. Using the same animal model, Ohta and coworkers demonstrated that perinatal exposure to constant light (24 L:0D) leads mice to have disrupted Per1 expression waveform in the developing SCN with desynchronization of the circadian rhythms of individual clock neurons within the SCN, but without reduction in the ability of single neurons to generate circadian rhythms (Ohta et al., 2005, 2006). Another group found that mice reared in constant light (24 L:0D) showed a higher locomotor activity rhythm amplitude and lower levels of vasoactive intestinal peptide (VIP) and arginine vasopressin (AVP) in the SCN compared to mice reared in constant darkness (0 L:24D) or 12 L:12D LD cycles (Smith and Canal, 2009). These results suggest that postnatal light experiences affect the SCN neuronal network that regulates seasonal adaptation by affecting the clock functions and output of SCN neurons. Recently, the same group found that mice raised in constant light showed stronger and shorter-period circadian rhythms of body temperature compared to mice raised in constant darkness and also displayed a greater tendency for depression-like behavior compared to 12 L:12D LD cycle-raised mice (Coleman et al., 2016). Moreover, compared to 12 L:12D LD cycle-raised mice, both constant-light and constant-dark-raised mice showed a decreased glucocorticoid receptor expression in the hippocampus and increased plasma corticosterone concentration at the onset of night. This demonstrates a possibility that the postnatal light environment induces long-term effects on the hypothalamic–pituitary–adrenal axis and circadian system leading to a depressive phenotype in adulthood.

These clinical and animal studies suggest that a 12 L:12D LD cycle would be safe as a NICU lighting cycle for the promotion of sleep and psychological development of preterm infants.

#### What light intensities are appropriate for light/dark cycles in NICUs?

Lighting recommendations by the American Academy of Pediatrics (AAP) and American College of Obstetricians and Gynecologists (ACOG) suggest that the light intensity of NICU illumination should range between 10 and 600 lux to allow continuous assessment of infants, as well as examination of skin color and perfusion (White, 2013; AAP Committee on Fetus and Newborn and ACOG Committee on Obstetric Practice, 2017). The Australian Health Infrastructure Alliance (AHIA) recommends an ambient light range of 100–600 lux [The Australian Health Infrastructure Alliance (AHIA), 2014]. Villalba et al. (2018) also stated that when using individual procedure lights, which are capable of reaching 2000 lux, care should be taken to avoid exposing the neonate’s eyes to high levels of light.

However, none of these guidelines specify optimal intensity ranges for varying gestational ages (Best et al., 2018). For light intensity during night, studies demonstrated that adult humans are responsive to a light intensity of approximately 30 lux and above when assessed by melatonin phase delay response to light and also responsive to a light intensity of approximately 50 lux and above when assessed by the acute melatonin suppressive effects of light (the phase change of melatonin circadian rhythm and suppression of plasma melatonin level have been used as the gold standard to evaluate the effects of light on biological clock) (St. Hilaire et al., 2012). The data indicate that human adults biologically recognize brightness at night when light intensity is 30–50 lux or above. The adult data suggest that the light intensity in NICUs at night should be lower than 30 lux, which preterm infants would not biologically perceive as brightness. The light sensitivity of preterm infants at each gestational age, however, has not been investigated. The light sensitivity of preterm infants can vary depending on their developmental stage. According to our own preterm study (Watanabe et al., 2013), 5 lux does not induce pupillary light reflex in preterm infants between 30 and 38 WGA, suggesting a light intensity of <5 lux, which was recommended for night NICUs by the American Academy of Pediatrics (AAP) and American College of Obstetricians and Gynecologists (ACOG), would be safe for preterm infants of all gestational ages in a NICU lighting environment (White, 2013; AAP Committee on Fetus and Newborn and ACOG Committee on Obstetric Practice, 2017).

For light intensity during daytime, an intensity between 30 and 440 lux would be safe for a NICU lighting environment. As earlier mentioned, adult data on phase change of daily melatonin rhythm suggest that preterm infants are likely to perceive daytime brightness at a light intensity of ≥30 lux. A safe brightness level during daytime would be 440 lux since a US multicenter study demonstrated that no clinical damage was detected in the eyes of preterm infants who were raised in constant light at an average light intensity of 447 lux (Reynolds et al., 1998).

#### What light wave lengths are appropriate for the light/dark periods in NICUs?

**Figure 1:**
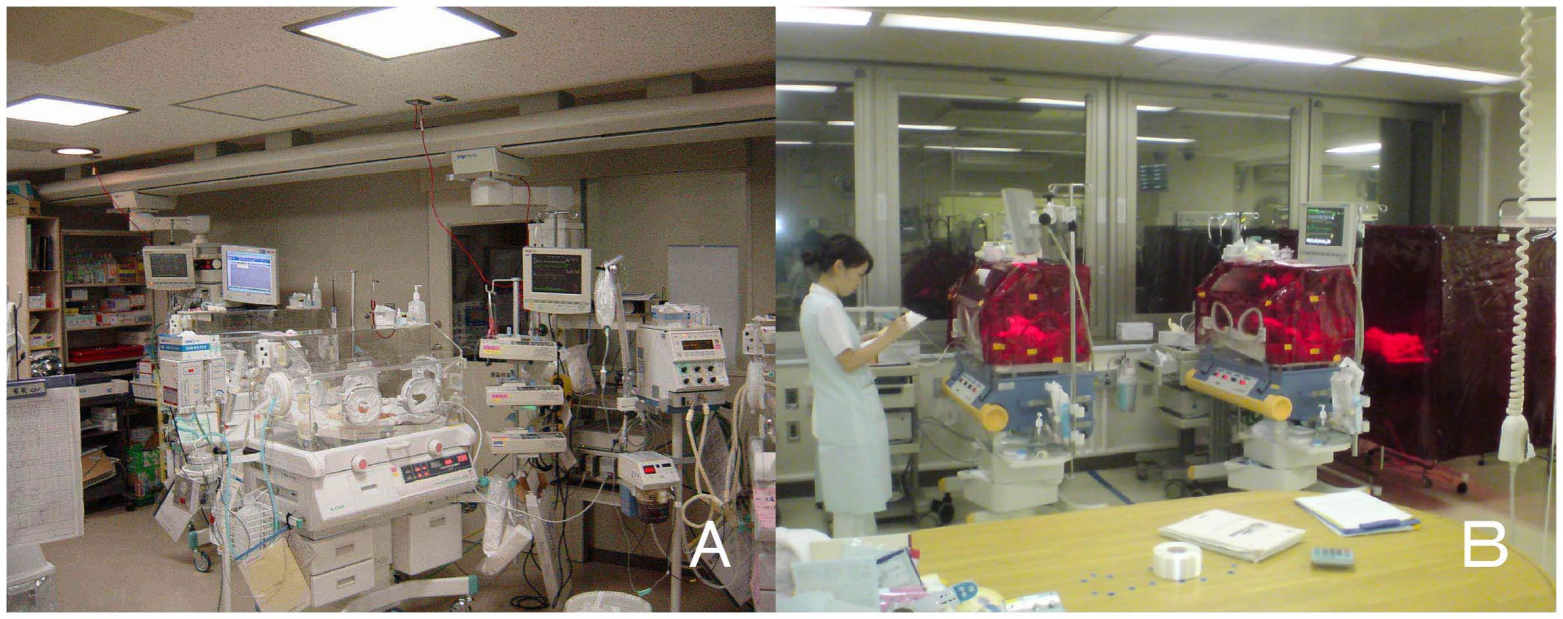
Bed-site illumination before and after introduction of a monochromatic light red filter. **(A)** In a former NICU, continuous lighting was selected to allow instant reaction to emergency situations such as respiratory and cardiovascular dysfunctions, brain hemorriage, and infections. The background lighting was generally between 50 and 600 lux. **(B)** Artificial night was achieved by covering the isolette or crib every night with a light filter, which specifically cuts the wavelength of light that the ipRGCs and rods of preterm infants detect. During daytime, the light filter was removed from the isolette (Watanabe et al., 2013).

Previous clinical studies indicate that the optimum light wavelengths for light/dark periods depend on the developmental stages of the preterm infants since the three types of visual photoreceptors in the retina (ipRGCs, rods, and cones) each start to function at a different time somewhere between approximately 26 WGA and 1 month after birth (where birth occurs at 40 WGA). Studies of the pupillary light reflex (PLR) of preterm infants revealed that only ipRGCs provide visual perception at the early stages of human development – at approximately 30 WGA – and contributes to the detection of changes in environmental light radiance (Robinson and Fielder, 1990; Hanita et al., 2009; Watanabe et al., 2013). Rods start to function at around 34 WGA and provides image detection (Figure 1; Watanabe et al., 2013; Ikeda et al., 2015). Cones begin to function 1 month after birth (where birth occurs at 40 WGA) (Field et al., 1982; Teller, 1997) and perform image and color detection.

**Figure 2:**
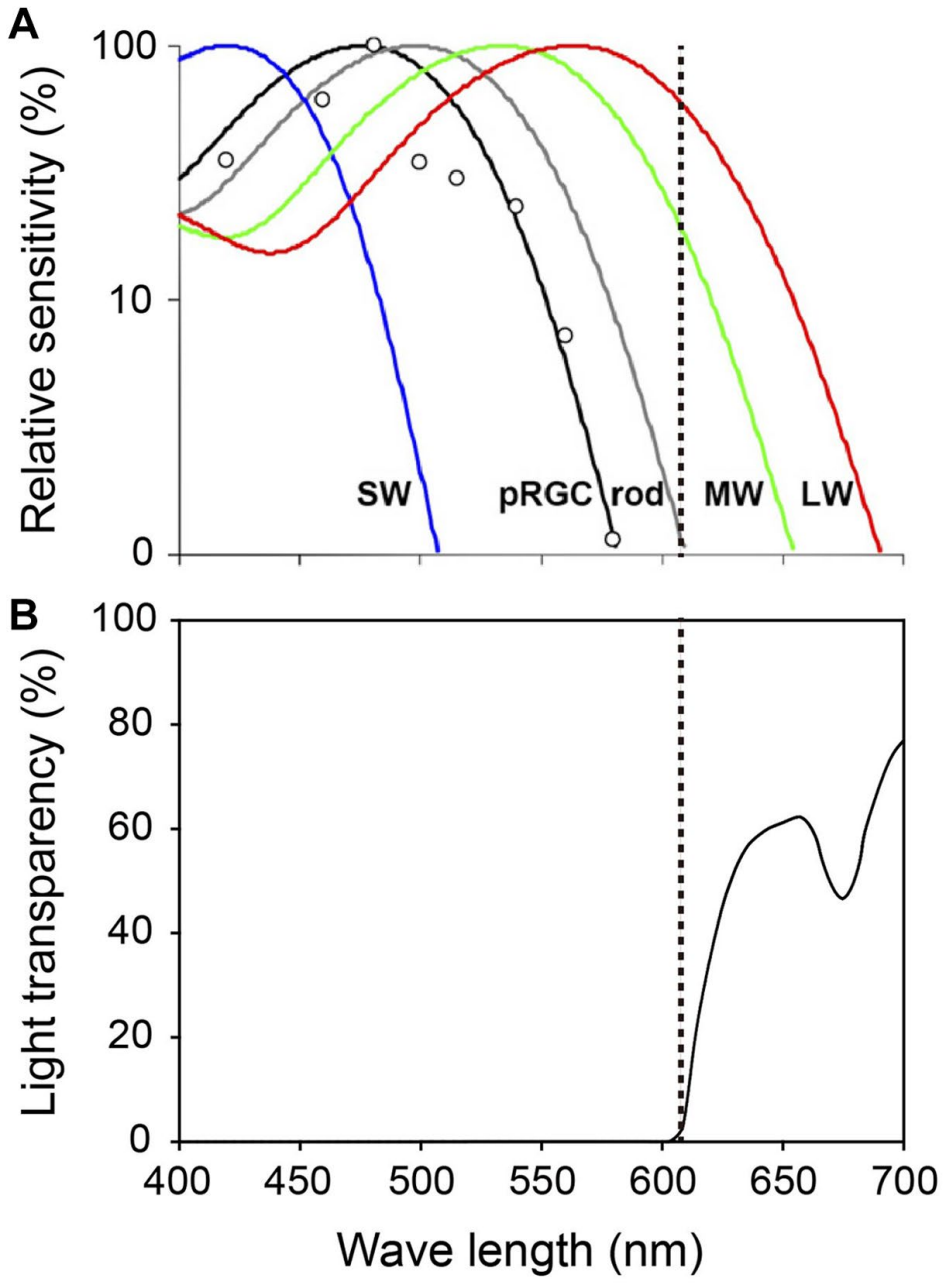
Spectral properties of human photoreceptors and spectral transmission characteristics of the red filter. **(A)** Spectral sensitivity of rods, cones and ipRGCs measured by pupil responses to light. Maximum light sensitivity wavelengths of human rods (R), S cones (SW), M cones (MW) and L cones (LW) are approximately 500 nm, 420 nm, 530 nm and 560 nm, respectively. The ipRGCs (depicted as pRGCs in the figure) exhibit their peak sensitivity at around 480 nm. The dashed line on the figure indicates 610 nanometers. Figure cited from Zaidi et al. (2007). White circles indicate the pupil responses to light in a blind woman. **(B)** Spectral transmission characteristics of the red filter in Figure 1B. Note that the red filter cut light of wave lengths <610 nm perceived by ipRGCs and rods, which function in preterm infants, while allowing wave lengths >610 nm perceived by adult MW and LW to pass through (Watanabe et al., 2013).

Adult studies have clarified the properties of the three types of photoreceptors and indicate that ipRGCs detect light of wave lengths up to 580 nm (maximum light sensitivity: 480 nm), rods detect wave lengths up to 610 nm (maximum light sensitivity: 500 nm), and cones detect wave lengths up to 690 nm (maximum light sensitivities of S, M, and L cones: 420, 530, and 560 nm, respectively) (Figure 2; Zaidi et al., 2007). A PLR study of preterm infants of 38 WGA, the age at which most preterm infants are discharged from hospital, demonstrated that they did not respond to 635 nm light but responded to 470 nm (Ikeda et al., 2015). This indicates that cones do not function at that age since 635 nm falls within the wave-length range normally detected by cones. However, since the preterm infants could respond to 470 nm light, this also indicates they are very likely to perceive light through ipRGCs and/or rods, which detect wave lengths up to 580 nm and 610 nm, respectively. Since ipRGCs starts to function earlier than rods and cones and convey light information well enough to control the SCN, the light wave length of <580 nm might be best for use in the design of NICU light–dark cycles.

### How can we entrain the biological clocks of young preterm infants who are not able to perceive light? - touch and sound may be effective ways of entraining preterms’ biological clocks

Since preterm infants are still in the process of developing the visual system and biological clock, further research should be carefully explored to understand their light sensitivity and entrainment (Hazelhoff et al., 2021; Polese et al., 2022). Light is the most powerful stimuli for the SCN, the biological clock, to synchronize to the environment. However, in the absence of light (i.e., constant darkness), the SCN of human adults can be synchronized by tactile and auditory cues. The physiological conditions of human adults in constant darkness are quite similar to the conditions of preterm infants less than 30 WGA, who do not yet fully respond to light. Human fetuses start to develop their tactile and auditory perceptions at around 16 and 28 WGA, respectively. Their tactile perception starts to function earlier than light perception and their auditory perception starts at approximately the same developmental age as light perception (Humphrey, 1964; Birnholz and Benacerraf, 1983; Lary et al., 1985; Schaal et al., 2004; Table 1).

**Table 1:** Developmental time line of human sensory systems and circadian entrainment.

Weeks gestational age (WGA)	Sensory systems	Circadian entrainment
16	Tactile perception^a^	
26	Possible SCN light responsiveness^b^	
28	Auditory perception^c^	
29	Olfactory perception^d^	
30	Pupil light reflex^e^	
33–42		Possible tactile entrainment^f^
38–43		Possible light entrainment^g^

a. Humphrey, 1964; b. Hao and Rivkees, 1999; c. Birnholz and Benacerraf, 1983, Lary et al., 1985; d. Schaal et al., 2004; e. Robinson and Fielder, 1990, f. Kuhn et al., 1991, White-traut et al., 1997, Ferber et al., 2002; g. Rivkees et al., 2004, Watanabe et al., 2013.

**Figure 3:**
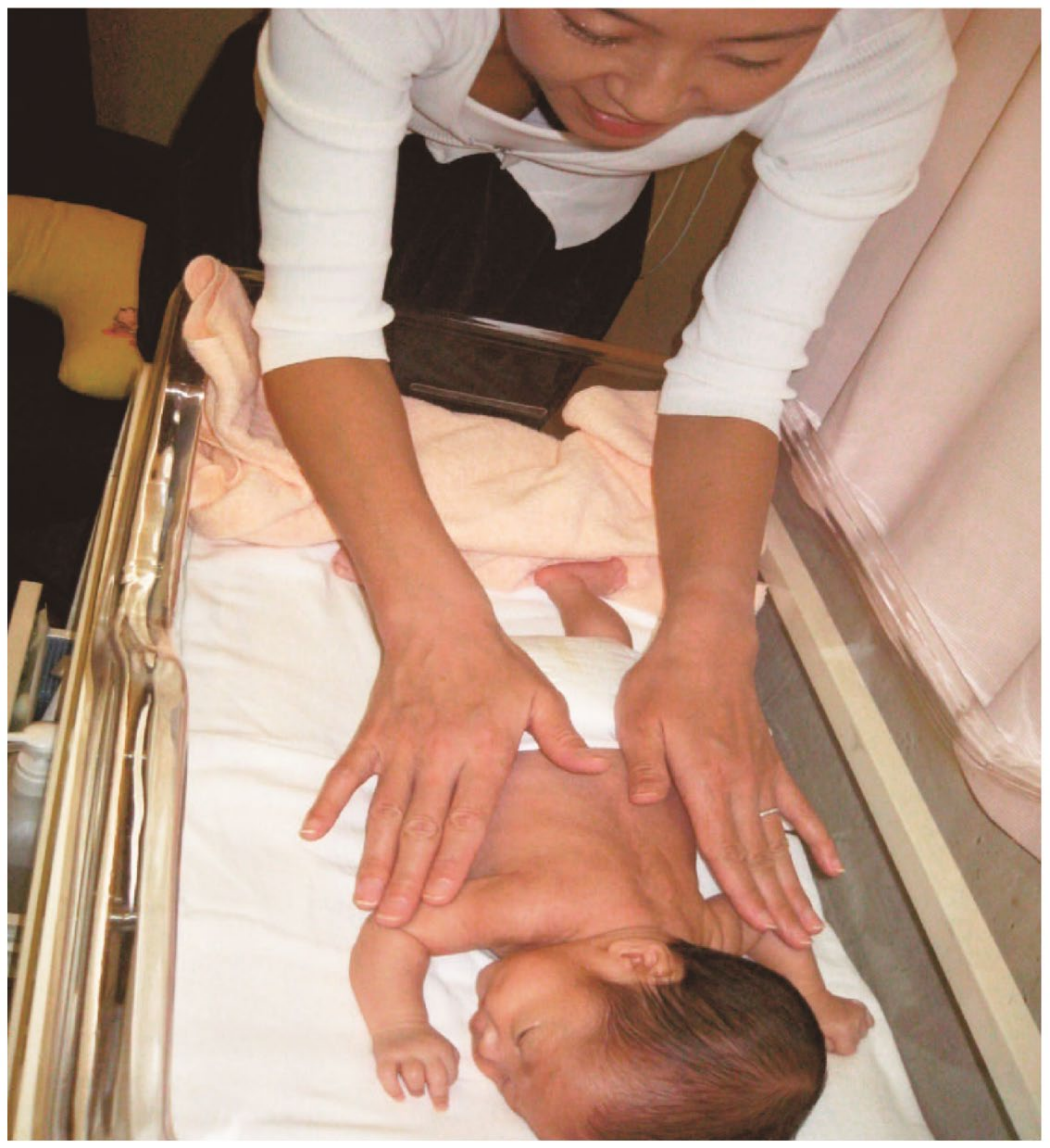
Preterm infant being massaged. Figure cited from Imura et al. (2014).

Despite few dedicated human neonate trials on the influence of tactile contact on the circadian clock, tactile stimulation has been medically performed on preterm infants in NICUs through massage therapy since the 1980s (Figure 3; Field et al., 2006; Badr et al., 2015). Randomized controlled trials (RCTs) on the effects of massage therapy in preterm infants confirm improved weight gain and shortened length of hospital stay (Badr et al., 2015; Niemi, 2017). Previous studies have reported that tactile stimulation leads to higher salivary concentration of both norepinephrine and epinephrine and an increase in the alert state (Kuhn et al., 1991; White-traut et al., 1997), suggesting that tactile stimuli may indeed contribute to the formation of circadian sleep/wake rhythms with more arousal during wake-time period of preterm infants of around 33–34 WGA. In a study of term infants equivalent to 40–42 WGA, massage therapy was reported to enhance their circadian rest-activity rhythms and elevated their nighttime excretion of urinary 6-sulphatoxymelatonin, the melatonin metabolite (Ferber et al., 2002). Massage therapy has also been reported to decrease infant crying and salivary cortisol, suggesting that tactile stimuli may also contribute to reducing psychophysiological stress in preterm infants. This is consistent with an animal study in which elevated cortisol levels caused by maternal deprivation were reduced in response to tactile stimulation (Pauk et al., 1986).

One means of improving the efficacy of massage therapy is to perform it with the infant in intermittent kangaroo position. It has been reported that massage therapy in intermittent kangaroo position leads to greater daily weight gain compared to massage therapy in incubators (Aldana Acosta et al., 2019). Kangaroo care is well known for decreasing mortality among preterm infants and improving rates of exclusive breast feeding in both developed and developing countries (Boundy et al., 2016). Tactile stimulation provided by kangaroo care, as well as continuous interaction with the parent, may also contribute to the organization of sleep–wake cyclicity and better modulation of the arousal system (Feldman et al., 2002). There is also a possibility that parental circadian body temperature supports the formation of the infant’s own circadian rhythms.

Auditory stimuli can also have a circadian synchronization effect on preterm infants. Auditory stimuli are known to influence the circadian rhythms of a variety of animals. For instance, the behavioral rhythms of the house sparrow (*Passer domesticus*) can be entrained by conspecific bird songs (Menaker and Eskin, 1966) and even by white noise (Reebs, 1989). Mice also show circadian responses to auditory cues, while other rodents like rats and hamsters do not (Davidson and Menaker, 2003). The efficacy of auditory stimuli entrainment thus varies among species. In human adults, presentation of an auditory stimulus at nighttime can phase shift circadian rhythms of melatonin and core body temperature (Goel, 2005), as well as synchronize activity rhythms in constant darkness but not in LD conditions (Davidson and Menaker, 2003). Preterm infants whose eyes are still immature (< 30 WGA) do not yet have matured ability to perceive light and therefore experience near-constant darkness. In the absence of visual cues, it is possible that such infants’ circadian systems can instead influenced by auditory stimuli.

Regarding sense of smell, no olfactory stimuli have been reported to phase-shift human behavioral or hormonal circadian rhythms. In animals, however, olfactory stimuli are known to affect the circadian clocks of a number of both invertebrate and vertebrate species. A study of social interaction effects on circadian rhythms suggests that olfactory signals can function as synchronizers in fruit flies (*Drosophila melanogaster*). Olfactory cues (possibly pheromonal) have also been implicated as synchronizers in a mammalian species, the degu (Octogon degus), a diurnal rodent. In addition, a mammary pheromone, aldehyde 2-methylbut-2-enal (2 MB2), was reported to act as a maternal olfactory cue to synchronize the circadian clock of artificially raised European newborn rabbits (Montúfar-Chaveznava et al., 2013).

Among the above mentioned three social cues of touch, sound, and smell, both tactile and auditory stimuli can phase-shift circadian clocks of preterm infants in normal lighting conditions: a light–dark cycle. Since tactile sensitivity is the first of the human senses to appear (at 16 WGA), appropriate tactile stimuli may be able to act as signals to synchronize the circadian clocks of preterm infants before they begin to perceive light at around 26–30 WGA. Auditory stimuli may also potentially support the stability of circadian rhythmicity once infants become sensitive to sound at approximately 28 WGA.

## Conclusion and near future goals

Preterm infants of approximately 22 WGA, the earliest age at which artificial ventilators are able to be applied, can be saved with medical treatments in Japan. Past literature suggests that the circadian rhythms of preterm infants of >26–30 WGA can be safely and effectively supported by LD cycles of 12 L:12D at a light intensity of >50 lux during daytime and < 5 lux during nighttime with wavelengths of up to 580 nm, which the ipRGCs of preterm infants, the first functioning photoreceptors in life, can detect.

The circadian rhythms of preterm infants of <30 WGA, however, are not likely to be fully assisted by LD cycles since their visual photoreceptors are still premature and do not yet function effectively. At such an age, tactile stimuli such as massage therapy in a circadian manner might be an effective way to entrain the circadian rhythms of preterm infants, leading to better body growth, since human tactile perception starts to function at 16 WGA, far before 26 WGA when ipRGCs start to function. In addition, auditory stimuli, such as parents’ voices may also entrain preterm infants’ circadian rhythms since auditory stimuli has been found to affect human circadian clocks in constant darkness, which preterm infants of <30 WGA experience due to immature visual function.

Moreover, a multisensory approach such as the combination of touch, sound and smell might be an important strategy for generating circadian rhythmicity in preterm infants more effectively in the future (Ferber et al., 2002; Neel et al., 2019). Animal neonate studies using rodents such as hamsters, mice and rats, clearly demonstrated that maternal multisensory stimuli such as touch, sound, smell and feeding phase-shifts neonatal circadian rhythms of behavior, hormones, and clock gene expressions. In one such study, newborn rats of mothers kept in an LD cycle were blinded immediately after birth and reared by foster mothers under either LD (LD blind pups) or a reversed light–dark (DL) cycle (DL blind pups). At postnatal day 6, significant phase differences were observed in the circadian gene expression rhythms of the SCN between the LD and DL blind pups, indicating that the two different maternal circadian behavioral patterns with multisensory stimuli in antiphase affected the circadian rhythms of the two different groups of blind pups, respectively, (Ohta et al., 2002). Recent studies on preterm and term infants have also suggested that not only circadian synchronization (entrainment) but also general brain functional connectivity can be affected by multisensory systems when they are exposed to various stimuli in extrauterine environments (Gatti et al., 2012; De Asis-Cruz et al., 2020; Polese et al., 2021; Pineda et al., 2021; Schmidt Mellado et al., 2022).

If, using circadian science, clinicians and researchers are able to redesign the presently employed protocol of kangaroo care, which is composed of parental touch, voice, and smell, the resulting modified form of kangaroo care may be a strong synchronizer for the circadian clock of preterm infants.

## Author contributions

TA, RF, MK, K-iT, HG, MH, SO, and HO conceived of the review. TA, RF, MK, HS, YY, K-iT, HG, YS, HAd, HAr, MH, SO, and HO wrote the paper. All authors contributed to the article and approved the submitted version.

## Funding

This work was supported by Grants-in-Aid for Scientific Research from the Ministry of Education, Culture, Sports, Science and Technology (to TA, RF, MK, YY, K-iT, HG, HAd, HAr, SO, and HO), from the Japan Science and Technology Agency (to HO), and also from the Uhehara Memorial Foundation (to HO).

## Conflict of interest

The authors declare that the research was conducted in the absence of any commercial or financial relationships that could be construed as a potential conflict of interest.

## Publisher’s note

All claims expressed in this article are solely those of the authors and do not necessarily represent those of their affiliated organizations, or those of the publisher, the editors and the reviewers. Any product that may be evaluated in this article, or claim that may be made by its manufacturer, is not guaranteed or endorsed by the publisher.
